# Supplementary material for: Variation in a Darwin Wasp (Hymenoptera: Ichneumonidae) Community along an Elevation Gradient in a Tropical Biodiversity Hotspot: Implications for Ecology and Conservation
Source: Insects. 2023 Nov 7;14(11):861. doi: 10.3390/insects14110861 (PMC10671876; doi:10.3390/insects14110861)
Supplement: Supplementary file 1 [file insects-14-00861-s001.zip › insects-2622983-supplementary/Supplementary Tables and Figures.pdf]

Table S1. Linear models of pimpline community metrics (response variable) against elevation at site level (n = 15). Model coefficients are given for the predictor variables, *significant predictor. Models within two AICc units of the model with the lowest AICc are in bold.

Response variable	Intercept	Elevation	Elevation^2	Elevation^3	Elevation^4	Model R^2	AICc
Abundance	109.64*	-0.0049				0.004	169.64
	**39.56**	**0.1752**	**-7.834 x 10^{-5}**			**0.284**	**168.49**
	**124.9**	**0.2458**	**3.844 x 10^{-4}**	**-1.371 x 10^{-7}***		**0.509**	**167.50**
	112.6	0.1577	2.22 3x 10^{-4}	-2.959 x 10^{-8}	-2.339 x 10^{-11}	0.511	173.28
Log $_{10}$ Species Richness	1.599*	-2.678 x 10^{-4}*				0.385	5.472
	**1.221***	**7.036 x 10^{-4}***	**-4.225 x 10^{-7}***			**0.670**	**-0.0589**
	**1.500***	**-6.7125 x 10^{-4}**	**1.088 x 10^{-6}**	**-4.476 x 10^{-10}**		**0.754**	**0.2096**
	1.398*	5.771 x 10^{-5}	-2.518 x 10^{-7}	4.414 x 10^{-10}	-1.935 x 10^{-13}	0.759	5.759
Log $_{10}$ Inverse Simpson’s Index (1/*D*)	**1.230***	**-4.056 x 10^{-4}***				**0.601**	**4.77**
	1.068*	1.043 x 10^{-5}	-1.089 x 10^{-7}			0.637	7.18
	0.9665*	5.137 x 10^{-4}	-7.34 x 10^{-7}	1.639 x 10^{-10}		0.644	11.53
	0.9057	9.503 x 10^{-4}	-1.537 x 10^{-6}	6.964 x 10^{-10}	-1.159 x 10^{-13}	0.645	17.32
Shannon Index	**3.240***	**-0.00087***				**0.621**	**26.44**
	**2.640***	**6.728 x 10^{-4}**	**-6.715 x 10^{-7}***			**0.731**	**25.13**
	2.739*	1.8 x 10^{-4}	-1.299 x 10^{-7}	-1.605 x 10^{-10}		0.732	29.71
	2.561*	1.462x10^{-3}	-2.488 x 10^{-6}	1.404 x 10^{-9}	-3.403 x 10^{-13}	0.734	35.42

Table S2. Bray-Curtis dissimilarity scores across the 15 sites.

Site	1	2	3	4	5	6	7	8
2	0.571	0						
3	0.677	0.542	0					
4	0.560	0.651	0.514	0				
5	0.818	0.752	0.703	0.698	0			
6	0.725	0.718	0.602	0.515	0.631	0		
7	0.746	0.815	0.701	0.628	0.763	0.651	0	
8	0.753	0.903	0.840	0.773	0.929	0.794	0.529	0
9	0.842	0.915	0.865	0.835	0.937	0.845	0.731	0.666
10	0.920	0.953	0.917	0.887	0.940	0.869	0.731	0.668
11	0.959	0.980	0.880	0.894	0.918	0.841	0.816	0.646
12	0.972	0.982	0.922	0.910	0.952	0.905	0.809	0.680
13	0.988	0.977	0.934	0.910	0.967	0.904	0.831	0.703
14	1.000	0.985	0.953	0.944	1.000	0.887	0.826	0.519
15	1.000	1.000	0.971	0.973	1.000	0.912	0.828	0.740

Table S2 continued

Site	9	10	11	12	13	14	15
10	0.421	0					
11	0.487	0.394	0				
12	0.456	0.416	0.273	0			
13	0.804	0.697	0.579	0.522	0		
14	0.715	0.640	0.485	0.564	0.556	0	
15	0.884	0.862	0.746	0.819	0.694	0.536	0

Table S3. Bray-Curtis dissimilarity scores between the 30 traps.

Trap	1A	1B	2A	2B	3A	3B	4A	4B
1A	0							
1B	0.675	0						
2A	0.607	0.639	0					
2B	0.670	0.711	0.566	0				
3A	0.723	0.640	0.605	0.586	0			
3B	0.755	0.788	0.667	0.617	0.627	0		
4A	0.624	0.738	0.656	0.695	0.587	0.648	0	
4B	0.784	0.630	0.787	0.714	0.623	0.571	0.489	0
5A	1.000	1.000	1.000	0.965	0.933	0.969	0.910	0.941
5B	0.719	0.886	0.893	0.728	0.833	0.636	0.780	0.717
6A	0.639	0.730	0.733	0.720	0.650	0.589	0.545	0.514
6B	0.781	0.943	0.893	0.827	0.786	0.682	0.670	0.674
7A	0.810	0.848	0.864	0.818	0.753	0.800	0.699	0.662
7B	0.750	0.691	0.833	0.785	0.710	0.750	0.695	0.606
8A	0.904	0.775	0.920	0.940	0.845	0.925	0.818	0.784
8B	0.863	0.696	0.908	0.911	0.785	0.918	0.820	0.743
9A	0.919	0.843	0.927	0.937	0.846	0.898	0.859	0.810
9B	0.920	0.908	0.932	0.944	0.931	0.946	0.908	0.895
10A	0.929	0.966	0.962	0.969	0.880	0.927	0.886	0.872

Table S3 continued

Trap	1A	1B	2A	2B	3A	3B	4A	4B
10B	0.924	0.957	0.984	0.946	0.947	0.949	0.937	0.913
11A	0.970	0.973	0.966	1.000	0.931	0.934	0.915	0.937
11B	0.967	0.953	0.983	0.986	0.916	0.878	0.933	0.881
12A	0.980	1.000	1.000	1.000	0.950	0.952	0.953	0.938

12B	0.946	1.000	0.981	0.969	0.893	0.926	0.899	0.885
13A	0.972	0.974	0.968	0.977	0.934	0.937	0.959	0.919
13B	1.000	1.000	1.000	0.982	0.931	0.950	0.951	0.903
14A	1.000	1.000	0.966	1.000	0.931	0.934	0.957	0.895
14B	1.000	1.000	1.000	1.000	0.974	0.976	1.000	0.953
15A	1.000	1.000	1.000	1.000	0.972	0.974	1.000	0.950
15B	1.000	1.000	1.000	1.000	0.967	1.000	1.000	1.000

Table S3 continued

Trap	5A	5B	6A	6B	7A	7B	8A	8B
5B	0.933	0						
6A	0.918	0.726	0					
6B	0.800	0.667	0.479	0				
7A	0.957	0.793	0.659	0.724	0			
7B	0.971	0.723	0.699	0.766	0.500	0		
8A	0.959	0.972	0.783	0.918	0.674	0.575	0	
8B	0.949	0.905	0.756	0.905	0.600	0.495	0.390	0
9A	0.986	0.914	0.845	0.914	0.750	0.752	0.657	0.766
9B	1.000	0.965	0.851	0.965	0.751	0.781	0.582	0.742
10A	1.000	0.981	0.852	0.922	0.733	0.790	0.557	0.768

Table S3 continued.

Trap	5A	5B	6A	6B	7A	7B	8A	8B
10B	1.000	0.902	0.872	0.934	0.728	0.815	0.645	0.786
11A	0.939	0.965	0.868	0.895	0.832	0.753	0.474	0.670
11B	0.978	0.929	0.848	0.876	0.863	0.856	0.621	0.836
12A	1.000	0.978	0.890	0.978	0.842	0.831	0.578	0.798
12B	1.000	0.921	0.867	0.941	0.828	0.816	0.600	0.782
13A	1.000	0.967	0.850	0.967	0.821	0.822	0.725	0.743

13B	0.968	0.977	0.886	0.954	0.851	0.857	0.733	0.789
14A	1.000	1.000	0.842	0.895	0.815	0.835	0.447	0.727
14B	1.000	1.000	0.881	0.958	0.855	0.841	0.493	0.719
15A	1.000	1.000	0.902	0.953	0.846	0.829	0.672	0.686
15B	1.000	1.000	0.959	1.000	0.935	0.914	0.878	0.846

Table S3 continued

Trap	9A	9B	10A	10B	11A	11B	12A	12B
9B	0.381	0						
10A	0.592	0.549	0					
10B	0.461	0.322	0.392	0				
11A	0.709	0.644	0.547	0.632	0			
11B	0.448	0.414	0.531	0.392	0.569	0		
12A	0.606	0.483	0.511	0.430	0.484	0.356	0	
12B	0.455	0.358	0.507	0.361	0.558	0.225	0.255	0
13A	0.799	0.770	0.673	0.690	0.563	0.583	0.464	0.574
13B	0.835	0.810	0.704	0.727	0.640	0.614	0.508	0.609
14A	0.733	0.661	0.585	0.664	0.333	0.586	0.527	0.577
14B	0.782	0.688	0.649	0.707	0.294	0.626	0.548	0.600
15A	0.853	0.806	0.780	0.818	0.511	0.723	0.718	0.753
15B	0.957	0.934	0.924	0.939	0.818	0.933	0.909	0.922

Table S3 continued

Trap	13A	13B	14A	14B	15A
13B	0.355	0			
14A	0.531	0.618	0		
14B	0.455	0.575	0.177	0	
15A	0.429	0.622	0.422	0.278	0
15B	0.838	0.903	0.818	0.750	0.667

Table S4. Correlation matrix of community properties and habitat variables at site level (n = 15). Values above and to the right the diagonal are values of Spearman's Rho, whilst those below and to the left are *P* values (asterisk and bold indicates if still significant after False Discovery Rate correction for multiple comparisons). For variable definitions see Methods.

	Abundance	Richness	Inverse Simpson's	Shannon	Elevation	Mean temp.
Abundance		**0.717**	0.250	0.357	-0.054	0.050
Richness	0.00264*		**0.751**	**0.866**	-0.554	0.566
Inverse Simpson's	0.368	0.00125*		**0.968**	**-0.793**	**0.811**
Shannon	0.192	0.00003*	<0.00001*		**-0.768**	**0.782**
Elevation	0.853	0.0322	0.000674*	0.001282*		**-0.993**
Mean temp.	0.863	0.0277	0.000386*	0.000902*	<0.00001*	
Max. temp.	0.863	0.0546	0.00177*	0.00238*	<0.00001*	<0.00001*
Min. temp.	0.812	0.0329	0.000435*	0.00128*	<0.00001*	<0.00001*
Temp. amp.	0.532	0.0724	0.00813*	0.0111*	0.0286	0.0275
Humidity	0.940	0.437	0.899	0.528	0.405	0.532
Litter dry mass	0.658	0.350	0.0834	0.126	0.541	0.482
% Litter moisture	0.773	0.125	0.000988*	0.00313*	<0.00001*	<0.00001*
Large Tree density	0.161	0.285	0.696	0.608	0.617	0.617
Small Tree density	0.602	0.404	0.834	0.756	0.955	0.869
Palm density	0.493	0.153	0.101	0.0950	0.166	0.137
Tree fern density	0.491	0.294	0.205	0.156	0.407	0.491
Bamboo cover	0.805	0.0441	0.000174*	0.000626*	$5.28x10^{-6}$*	$6.67x10^{-6}$*
Fern cover	0.577	0.0308	0.00133*	0.00177*	0.000508*	0.000589*
Herb cover	0.150	0.00546*	0.00336*	0.000956*	0.212	0.204
Epiphyte density	0.825	0.0876	0.000209*	0.00124*	$5.63x10^{-5}$*	$2.35x10^{-5}$*

Liana density	0.775	0.0486	0.0549	0.0311	0.0172	0.0145*

Table S4 continued

	Max. temp.	Min. temp.	Temp. amp.	Humidity	Litter dry mass	% Litter moisture
Abundance	-0.043	0.068	-0.175	0.021	0.125	0.082
Richness	0.505	0.552	-0.477	0.217	0.260	-0.414
Inverse Simpson’s	**0.754**	**0.807**	**-0.668**	0.0357	0.464	**-0.779**
Shannon	**0.739**	**0.768**	**-0.646**	0.177	0.414	**-0.725**
Elevation	**-0.975**	**-0.986**	0.571	-0.232	-0.171	**0.889**
Mean temp.	**0.982**	**0.993**	-0.575	0.175	0.196	**-0.900**
Max. temp.		**0.971**	-0.511	0.248	0.100	**-0.893**
Min. temp.	<0.00001*		-0.618	0.127	0.175	**-0.907**
Temp. amp.	0.0543	0.0163		-0.043	-0.400	**0.621**
Humidity	0.372	0.652	0.879		-0.320	0.030
Litter mass	0.724	0.532	0.141	0.245		-0.311
Litter moisture	<0.00001*	<0.00001*	0.0156*	0.914	0.259	
Large Tree density	0.336	0.599	0.221	0.653	0.0546	0.599
Small Tree density	0.746	0.894	0.805	0.899	0.311	0.498
Palm density	0.162	0.0890	0.00874*	0.423	0.763	0.264
Tree fern density	0.407	0.491	0.330	0.0463	0.407	0.407
Bamboo cover	$3.31x10^{-5}$*	$3.21x10^{-6}$*	0.000694*	0.342	0.512	0.000116*
Fern cover	0.000982*	0.000618*	0.0266	0.843	0.160	0.00223*
Herb cover	0.276	0.258	0.0849	0.680	0.00587*	0.154
Epiphyte density	$8.63x10^{-5}$*	$1.03x10^{-5}$*	0.000697*	0.737	0.145	0.000197*
Liana density	0.0149*	0.0138*	0.0359	0.0977	0.800	0.117

Table S4 continued

	Large tree density	Small Tree density	Palm density	Tree fern density	Bamboo cover	Fern cover
Abundance	0.381	0.147	0.192	0.193	-0.070	-0.157
Richness	0.295	0.232	0.388	0.290	-0.526	-0.557
Inverse Simpson's	0.110	0.059	0.439	0.347	**-0.821**	**-0.748**
Shannon	0.144	0.088	0.447	0.386	**-0.779**	**-0.736**
Elevation	0.141	0.016	-0.377	-0.231	**0.899**	**0.786**
Mean temp.	-0.141	-0.047	0.402	0.193	**-0.895**	**-0.781**
Max. temp.	-0.267	-0.091	0.380	0.231	**-0.864**	**-0.761**
Min. temp.	-0.148	-0.038	0.454	0.193	**-0.906**	**-0.779**
Temp. amp.	-0.336	0.070	-0.650	-0.270	**0.775**	0.570
Humidity	-0.126	0.036	0.224	0.521	-0.264	-0.056
Litter mass	0.505	-0.281	0.085	-0.231	-0.184	-0.382
Litter moisture	0.148	0.190	-0.308	-0.231	**0.833**	**0.725**
Large Tree density		0.373	-0.042	-0.234	0.0411	-0.098
Small Tree density	0.170		-0.097	-0.135	0.064	-0.070
Palm density	0.882	0.731		0.199	-0.579	-0.228
Tree fern density	0.401	0.631	0.476		-0.439	-0.078
Bamboo cover	0.884	0.821	0.0238	0.101		**0.734**
Fern cover	0.729	0.803	0.413	0.783	0.00186*	
Herb cover	0.118	0.962	0.582	0.259	0.111	0.0117*
Epiphyte density	0.941	0.703	0.00595*	0.681	$7.63x10^{-6}$*	0.00175*
Liana density	0.761	0.334	0.00678*	0.490	0.0128*	0.234

Table S4 continued

	Herb cover	Epiphyte density	Liana density
Abundance	-0.391	0.063	0.081

Richness	**-0.678**	-0.456	0.517
Inverse Simpson's	**-0.705**	**-0.816**	0.505
Shannon	**-0.762**	**-0.751**	0.557
Elevation	0.342	**0.852**	-0.603
Mean temp.	-0.348	**-0.871**	0.616
Max. temp.	-0.301	**-0.841**	0.614
Min temp.	-0.312	**-0.887**	0.620
Temp amp.	0.459	**0.775**	-0.544
Humidity	-0.116	-0.095	0.444
Litter dry mass	**-0.674**	-0.395	-0.072
% Litter moisture	0.387	**0.818**	-0.423
Large tree density	-0.421	0.021	0.086
Small Tree density	0.014	0.108	0.268
Palm density	-0.155	**-0.673**	**0.665**
Tree fern density	-0.311	-0.116	0.193
Bamboo cover	0.429	**0.893**	**-0.624**
Fern cover	**0.631**	**0.736**	-0.327
Herb cover		0.404	-0.140
Epiphyte density	0.135		**-0.659**
Liana density	0.619	0.00752*	

Table S5. Correlation matrix of community properties and habitat variables at trap level (n = 30). Values above and to the right the diagonal are values of Spearman's Rho, whilst those below and to the left are *P* values (asterisk and bold indicates if still significant after False Discovery Rate correction for multiple comparisons). For variable definitions see Methods text.

	Abundance	Species richness	Inverse Simpson's index	Shannon index	Elevation	Litter dry mass
Abundance		**0.757**	0.289	**0.460**	-0.080	0.072
Species richness	1.29x10^{-6}*		**0.720**	**0.866**	**-0.516**	0.135
Inverse Simpson's index	0.121	7.33x10^{-6}*		**0.954**	**-0.742**	0.293
Shannon index	0.0106*	6.62x10^{-10}*	< 0.0001*		**-0.728**	0.194
Elevation	0.676	0.00354*	2.65x10^{-6}*	5.21x10^{-6}*		-0.069
Litter dry mass	0.705	0.477	0.116	0.304	0.717	
% Litter moisture	0.657	0.0332	7.51x10^{-6}*	7.21x10^{-5}*	2.89x10^{-10}*	0.346
Large tree density	0.447	0.626	0.950	0.937	0.302	0.105
Small tree density	0.655	0.705	0.762	0.830	0.742	0.876
Palm density	0.148	0.0133	0.0148*	0.0125*	0.0691	0.519
Tree Fern density	0.499	0.498	0.436	0.478	0.478	0.0774
Bamboo cover	0.831	0.0313	1.27x10^{-5}*	0.000277*	3.74x10^{-9}*	0.399
Fern cover	0.898	0.0621	0.00530*	0.00951*	0.00511*	0.759
Herb cover	0.0647	0.00634*	0.00446*	0.00433*	0.204	0.0534
Liana density	0.596	0.220	0.128	0.138	0.112	0.974
Epiphyte density	0.659	0.0719	0.000372*	0.00339*	0.000390*	0.000390*

Table S5. continued

	% Litter moisture	Large tree density	Small tree density	Palm density	Tree Fern density	Bamboo cover
Abundance	0.085	0.144	0.085	0.271	0.128	0.041
Species richness	-0.390	0.093	0.072	**0.447**	0.129	-0.394
Inverse Simpson's index	**-0.735**	0.012	-0.058	**0.441**	0.148	**-0.707**
Shannon index	**-0.672**	0.015	-0.041	**0.451**	0.135	**-0.618**
Elevation	**0.874**	0.195	0.063	-0.336	-0.135	**0.847**
Litter dry mass	-0.178	0.302	-0.030	0.122	-0.327	-0.160
% Litter moisture		0.138	0.191	-0.294	-0.109	**0.798**
Large tree density	0.466		0.224	0.049	-0.150	0.119
Small tree density	0.311	0.233		-0.058	0.071	0.050
Palm density	0.115	0.799	0.760		0.109	**-0.451**
Tree Fern density	0.566	0.428	0.710	0.568		-0.293
Bamboo cover	1.32×10^{-7}*	0.532	0.793	0.0125*	0.116	
Fern cover	0.00162*	0.686	0.622	0.629	0.652	0.0216
Herb cover	0.0893	0.0227	0.823	0.460	0.348	0.0925
Liana density	0.356	0.673	0.00881*	0.340	0.0648	0.211
Epiphyte density	1.39×10^{-5}*	0.256	0.0865	0.00454*	1.00	0.000236*

Table S5. continued

	Fern cover	Herb cover	Liana density	Epiphyte density
Abundance	-0.024	-0.342	0.101	0.084
Species richness	-0.345	**-0.487**	0.231	-0.333
Inverse Simpson's index	**-0.496**	**-0.505**	0.284	**-0.607**
Shannon index	**-0.466**	**-0.506**	0.278	**-0.518**
Elevation	**0.498**	0.238	-0.296	**0.606**
Litter dry mass	-0.058	-0.356	0.006	-0.160
% Litter moisture	**0.551**	0.316	-0.175	**0.705**
Large tree density	-0.077	-0.415	0.080	0.214
Small tree density	0.094	-0.043	**0.470**	0.318
Palm density	-0.092	-0.140	0.181	**-0.504**
Tree Fern density	-0.086	-0.177	0.341	0.00
Bamboo cover	0.418	0.313	-0.235	**0.623**
Fern cover		**0.490**	0.017	**0.590**
Herb cover	0.00597*		-0.171	0.110
Epiphyte density	0.928	0.367		0.021
Liana density	0.000603*	0.564	0.910	

Table S6. Principal Component weightings for the abiotic variables at trap level (n = 30), along with initial eigenvalues and % variance explained. For variable explanations and units, see Methods text. Variables were scaled prior to analysis.

Variable	PC1	PC2	PC3	PC4
Elevation	0.427	-0.0518	0.200	-0.129
Dry litter mass	-0.101	-0.447	0.321	0.157
% Litter moisture	0.424	0.0129	0.0136	-0.0104
Large tree density	-0.0385	-0.572	0.0867	0.0160
Small tree density	0.149	-0.428	-0.455	0.0798
Tree fern density	-0.121	0.111	-0.456	-0.491
Bamboo ground cover	0.421	0.0742	0.135	-0.0546
Fern ground cover	0.385	-0.0613	-0.170	0.214
Herb ground cover	0.226	0.453	-0.103	0.445
Epiphyte density	-0.0610	-0.0833	-0.591	0.137
Liana density	0.403	-0.230	-0.151	-0.00819
Palm density	-0.216	-0.0199	-0.066	0.667
Initial eigenvalue	4.133	2.026	1.676	1.078
% variance	34.44	16.88	13.97	8.98
% cumulative variance	34.44	51.32	65.29	74.28

Table S7. Best models of pimpline community properties against the abiotic variable Principal Components (PC) in Table S6. Best models were selected on the basis of AICc scores. *=significant predictor

Response variable	Intercept	Predictor variables		Model R^2
Abundance	65.73*	+4.205 x PC1	-3.436 x PC1*	0.225
Log_{10} Richness	1.237*	-0.0568 x PC1	-0.0361 x $PC1^2$*	0.547
Log_{10}(1/D)	0.711*	-0.134* x PC1*	-0.0415 x PC2	0.691
Shannon Index	2.021*	-0.297 x PC1*		0.634

Figure S1.

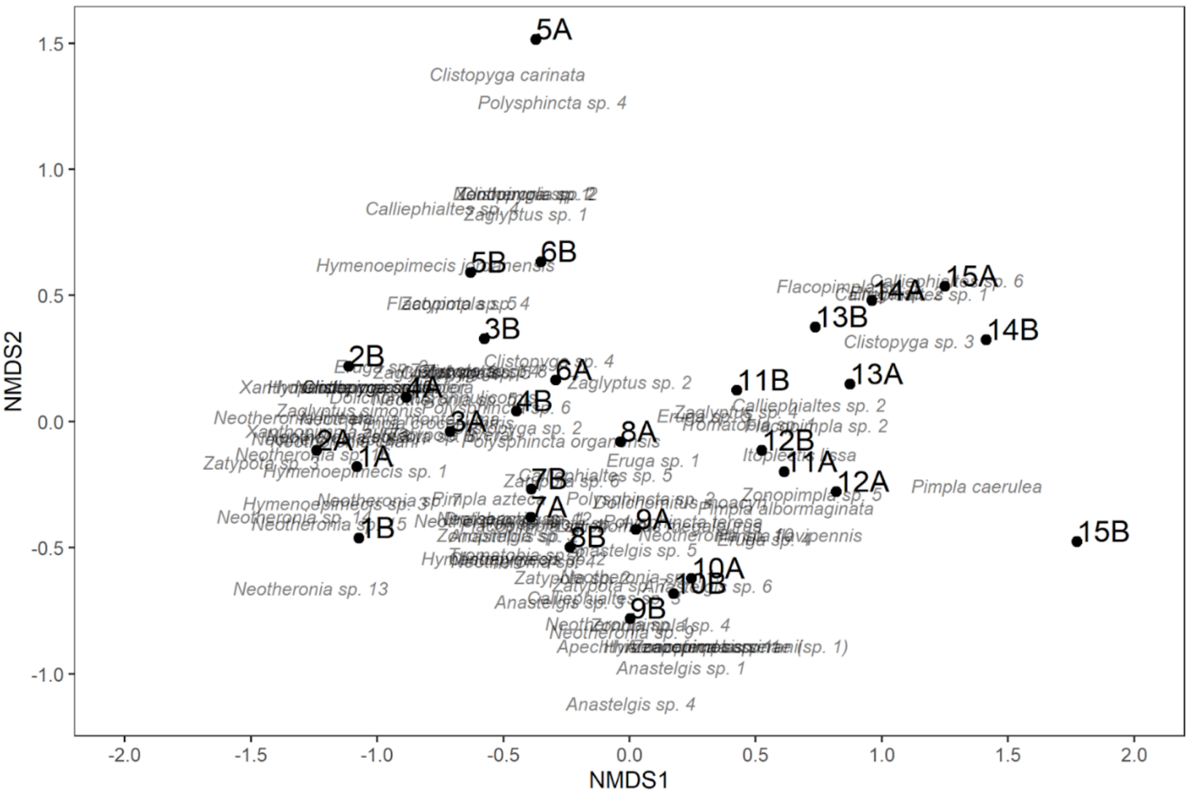

Figure S1. Non-metric Multidimensional Scaling (NMDS) analysis of the pimpline community at the trap level. Black numbers and points indicate the 30 traps at 15 sampling sites going from bottom of the mountain (1) to top (15). Species are in grey, small lettering.

Figure S2.

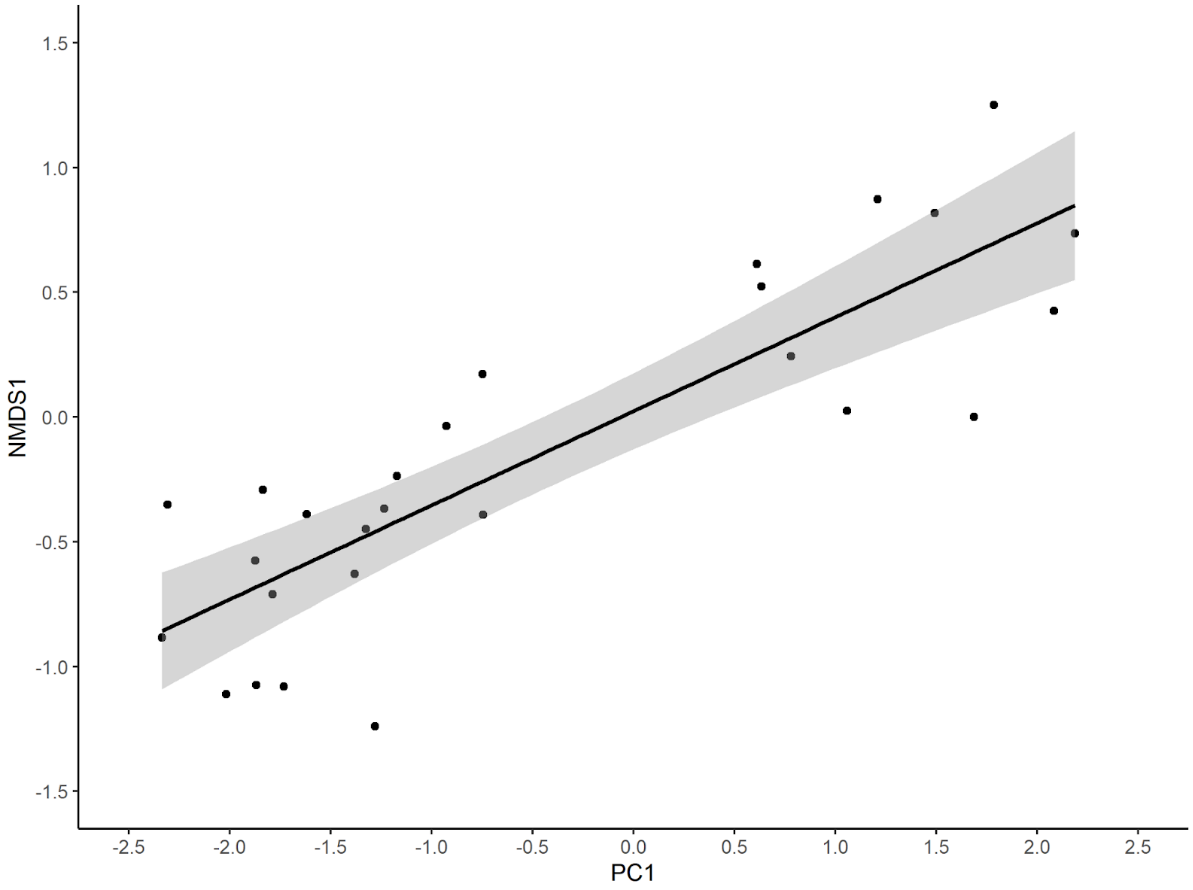

Figure S2. Pimplinae community composition, as measured by the first axis of a Non-Metric Multidimensional Scaling analysis (NMDS1, Figure S1) across 30 traps, against the first Principal Component (PC1) of the habitat variables at those traps (Table S6). The line is the linear regression (± 95%CI in grey). The figure demonstrates that pimpline community composition is strongly associated with differences in habitat characteristics.
